# Soft Modular Robotic Cubes: Toward Replicating Morphogenetic Movements of the Embryo

**DOI:** 10.1371/journal.pone.0169179

**Published:** 2017-01-06

**Authors:** Andrea Vergara, Yi-sheng Lau, Ricardo-Franco Mendoza-Garcia, Juan Cristóbal Zagal

**Affiliations:** 1 Departamento de Ingeniería Mecánica, Universidad de Chile, Santiago, Chile; 2 Escuela Universitaria de Ingeniería Mecánica, Universidad de Tarapacá, Arica, Chile; University of Vermont, UNITED STATES

## Abstract

In this paper we present a new type of simple, pneumatically actuated, soft modular robotic system that can reproduce fundamental cell behaviors observed during morphogenesis; the initial shaping stage of the living embryo. The fabrication method uses soft lithography for producing composite elastomeric hollow cubes and permanent magnets as passive docking mechanism. Actuation is achieved by controlling the internal pressurization of cubes with external micro air pumps. Our experiments show how simple soft robotic modules can serve to reproduce to great extend the overall mechanics of collective cell migration, delamination, invagination, involution, epiboly and even simple forms of self-reconfiguration. Instead of relying in complex rigid onboard docking hardware, we exploit the coordinated inflation/deflation of modules as a simple mechanism to detach/attach modules and even rearrange the spatial position of components. Our results suggest new avenues for producing inexpensive, yet functioning, synthetic morphogenetic systems and provide new tangible models of cell behavior.

## Introduction

Cells are the fundamental building block of living organisms. During morphogenesis, cells are able to contract, change their intercellular adhesion forces and even migrate, organizing themselves into different tissues that ultimately give rise to more complex structures and organs [1,2]. Researchers have tried to build modular self-reconfigurable robots imitating the capacity of cells to construct systems of varying morphology and function [3]. The development of this type of robots might result in more versatile and robust machines, capable of adapting their shape and function to account for new tasks, circumstances and even recover after damage [4]. Modular robots have been constructed using rigid materials, with cell-resembling docking units often carrying computation, sensing, actuation and energy storage capabilities, and have demonstrated self-reconfiguration, and even self-replication abilities under well-controlled experimental conditions [5–7].

**Fig 1:**
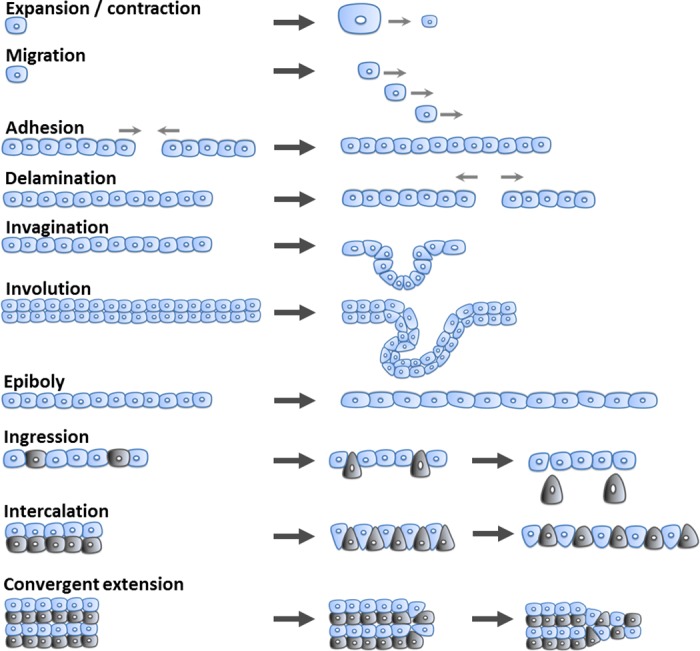
Outline of some of the fundamental cell behaviors that take place during morphogenesis. During expansion/contraction cells change their volume. During migration cells are able to travel to different locations. Cell adhesion involves the capability of cells to bind to other cells or substrate. Cell delamination involves splitting apart groups of cells. Cell invagination is a type of folding that creates a pocket. Cell involution is the generation of an inward curvature that results in a new underlying layer. Cell epiboly involves spreading of cell layers. During ingression cells detach from a main structure to migrate. During intercalation cells from different rows interpolate creating longer but thinner arrays. Convergent extension involves cells that converge in one direction to achieve extension in another perpendicular orientation.

While cells are inherently soft, current rigid implementations of modular robots fail at reproducing fundamental cell behaviors that require elements to shrink, squeeze and stretch while controlling connections to other units [8,9]. These capabilities are especially observed during the morphogenetic movements of gastrulation, the initial shaping stage of the embryo [10]. During this process, cells exhibit several mechanical behaviors (see Fig 1). Cells are able to expand and contract, migrate, attach to each other (cell adhesion), and detach from each other (cell delamination). Sheets of cells are able to bend inward (invagination), roll inward (involution), spread by thinning (epiboly) and other mechanically complex behaviors like detach and migrate freely (ingression) create longer but thinner arrays (intercalation), and even converge and extend (convergent extension). These set of behaviors allow the generation of infinite variation of living shapes, ranging from the primordial primitive streak to complex structures of intricate organs such as the heart. Mechanical forces and behavior result in biochemical changes that ultimately define function and structure of the cell [11]. Creating modular machines with the capability to replicate these movements will serve to better understand the way nature creates shape as well as to advance toward artificial systems that can grow and develop.

Recently a door to soft robotics was opened, and the new field is rapidly expanding with results on new soft actuators, soft sensors and intelligent soft mechanisms [12]. These robots are usually implemented with rubber, silicone or deformable material and are expected to overcome some of the limitations of current rigid robotics. Soft robots are expected to be more compliant, flexible, robust, light, stable, cheap and even simpler. For example, while many components are today required for the implementation of a joint or a linear actuator, only one soft structure might be required using soft robotic technologies [13,14]. Soft robots can manipulate delicate objects, conform to their surroundings, move in cluttered environments, and exhibit dramatic shape deformation [15].

This paper describes the design and fabrication of soft modular robotic cubes based on magnetic adhesion and pneumatic actuation. The parsimonious design of robotic units allows complex group behavior that resembles, to a large extent, some of the key morphogenetic movements of living cells. We demonstrate how soft modules can autonomously and purposively be detached and even self-reconfigured without relying on unit-embedded active undocking mechanisms but instead using the coordinated inflation of modules. We also characterize the mechanical behavior of modules and design walking controllers for three modular configurations. Finally we characterize their behavior in reality and simulation.

## Related Work

Onal and Rus introduced the concept of soft modular robots to literature [16]. They created a soft actuator made of two silicone halves, a non-extensible and an extensible one, which performed two-dimensional motion by bending in one direction when used alone or two directions when glued back-to-back to another actuator. The authors followed a modular approach by bonding actuators into serial and parallel configurations that, in combination with control sequences of electro-pneumatic valves, enabled different locomotion modes. Germann *et al*. proposed an active electrostatic connection mechanism for joining extremely lightweight soft modules [17]. On a different work his group also studied how chains made of soft ring-shaped limbs can display predictable folding behaviors when released over a flat surface [18]. They showed how chains constructed with different softness presets would lead to different curvilinear shapes when retracted. Kwok *et al*. designed a magnetic connector to join soft robots with hard components [19]. Their device uses an integrated expansive bladder to allow remote disassembly. Morin *et al*. proposed techniques for fabricating inflatable cubes from thin elastomeric tiles [20]. The use of double-tapper dovetails served at increasing the contact area before gluing tiles by their edges. They manually arranged cubes into different configurations using soft peg/recess surface connectors. Locomotion capabilities of preassembled soft modular systems have been studied in simulation and real implementations of chained inflatable spheres [21,22]. Rus and Vona introduced rigid modular robots able to achieve two-dimensional self-reconfiguration thanks to expansion/contraction of their flat faces [23]. Before us, Yu *et al*. visualized the potential of imitating fundamental cell movements with modular robots [24]. To validate their concept, they built the Morpho modular robot whose modules were linear actuators made of rigid materials covered with fabric. The authors combined the modules into assemblies that resulted in quick changes of shapes that they called “self-deformation”. Our work presents a new soft modular system that is able to autonomously self-reconfigure and reproduce various important cell behaviors. The design uses permanent magnets for inter-module self-aligned bonding and relies on simple coordinated inflation of modules to achieve remote assembly/disassembly.

## Design and Construction

**Fig 2:**
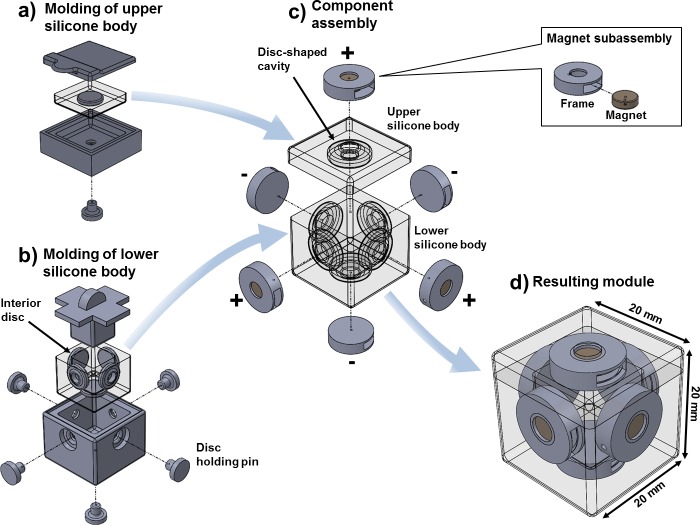
Fabrication of soft robotic modules. The process begins by producing two silicone bodies using multi-part 3D printed molds: the upper body (a) and the lower body (b). Removable pins hold the interior discs used to create cavities on each face. The resulting silicone bodies are glued together and magnet subassemblies are introduced inside the resulting disc-shaped cavities (c). The insert shows how magnets are introduced inside a wrapping frame to enlarge contact surface with silicone and improve bonding. The resulting module is finally shown in (d).

Our design was made with strong actuation, simplicity, and lightness in mind. We were focused on producing a system that reproduces the general aspects of cell-environment interaction rather than the complex physics of cell motility, adhesion or expansion. Simple inspection of gastrulation in drosophila [9] led us to choose three basic requirements for modules: They should (1) allow control of expansion/contraction, (2) adapt their shape to fit surrounding space and (3) have the ability to attach and detach to each other. We used a silicon elastomer (Ecoflex 00–30, Smooth-on, http://www.smooth-on.com) to fabricate the robotic modules with soft lithography [25]. The silicone is very soft and strong allowing for up to 900% strain before fracture. Fig 2 shows the fabrication process. Modules are 20×20×20mm (8cc) elastomeric cubes with a hollow core to enable pneumatic actuation by inflation, and a disc-shaped cavity on every face to hold Neodymium cylindrical permanent magnets (Duramag 3000 Gauss NdFeB Neo magnet, 6mm dia × 2mm thk) that serve for docking (Fig 2C and 2D). Rigid 3D-printed frames were used to avoid magnets from collapsing toward the center of the cubes, to provide a smooth transition between hard magnets and extremely soft silicone, as well as to improve bonding by increasing the contact area between the different materials. Frames fit tightly inside the disc-shaped holes of the soft body as shown in Fig 2D. The frames and required molds were 3D printed with photopolymer resin (RGD240, Stratasys, http://www.stratasys.com) using a high resolution 3D printer (ObJet 30, Stratasys, http://www.stratasys.com). The liquid polymer precursor was mixed and then poured into the 3D printed molds shown in Fig 2A and 2B.

The docking system results in homogeneous modules with gendered connecting faces, where three faces release a north-pole magnetic field and the other three a south-pole field (Fig 2C). The induced soft lattice structure constraints each module face to find a reversed polarity face in front. Modules can aggregate into arbitrary 3D shapes as long as their magnetic orientation matches the preexisting cubic lattice orientation and inter-module connection strength allows shape preservation [26]. Connection strength evaluations (S3 Appendix) indicated that up to eight modules can be vertically suspended on a single column and three modules cantilever. Pneumatic actuation and magnetic docking are compatible choices with the soft nature of the modules as they lack of internal mechanisms that would interfere with the elasticity otherwise. The size of modules was chosen to be as small as possible to simplify the scalability of the system.

The fabrication also relies in two multi-part molds. The first served for casting the upper body (Fig 2A) of a cube and the second for producing the lower body (Fig 2B) of a cube. These bodies are glued together using the same silicone resin that makes up the body. Curing liquid polymer on molds takes 20 minutes at 60°C; a process which otherwise might take up to four hours at room temperature. Magnets are then inserted on each face and covered with a sealing drop of silicone that prevents them from being pulled apart from the assembly. A final curing stage is executed during additional 10 minutes at 60°C. The final step consists on producing a small air inlet on one vertex of the cube and then introducing a 3mm airline. The resulting cube weights 10 grams (S3 Fig shows photo series of the manufacturing process).

Actuation is achieved by volumetric changes induced by computer-controlled pressurization of modules. Each module is connected to a dedicated pneumatic line driven by a miniature diaphragm air compressor (Thinker, 60 kPa pressure, 60 mL/min air flow) for pressurization and a solenoid valve (12v, 26 kPa max pressure, normally closed) for pressure relief. The electro-pneumatic setup (see S1 Appendix and S4 Fig) considers independent circuits for each module. The activation signals of air pumps and relief valves are driven by an Arduino Leonardo (http://www.arduino.cc). The low current signals were amplified with 2N2222 transistors.

## Experiments

The following is a description of the different behaviors that where reproduced with the coordinated actuation of the soft modules:

### Expansion/Contraction

**Fig 3:**
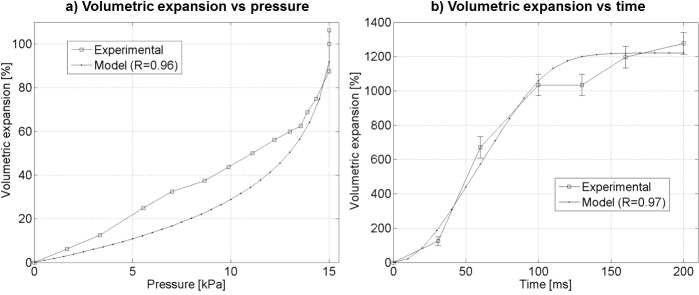
Volumetric Response of Modules. a) Volumetric expansion vs pressure. b) Instantaneous volumetric expansion response to a 138 kPa pressure impulse showing a rapid expansion of the module. Models described on Eqs 1 and 2 are fitted to the data.

We characterized the capability of soft modules to expand and contract when controlling their internal pressurization. We measured the volumetric expansion of cubes as function of their internal pressure (see Fig 3A). An expansion of 106% respect to initial external volume results when applying a pneumatic pressure of 15 kPa. We also measured the dynamic transient response to a 137.9 kPa impulse (see Fig 3B) applied to the input hose over a very short period of time. The figure shows how a rapid volumetric expansion (1277%) can be achieved after 200 ms.

Eq 1 models the normalized volumetric expansion as function of the static pressure *P*_*0*_ inside a module. The constant Δ*V*_*τ*_ describes the volumetric expansion at which accelerated expansion takes place, and *P*_*max*_ (*P*_*0*_ < *P*_*max*_) is the maximum pressure which can be applied to a module before failure.

$$\Delta V_{chamber}\left(P_{o}\right)=\Delta V_{\tau }∙ln\left(\frac{P_{max}}{P_{max}-P_{o}}\right)$$ (1)

Eq 2 adds time dependency to the same model by considering the dynamics required to build up pressure inside a module, *P*_*0*_ = *P*_*i*_ (1 –*e*^(-*t*/*RC*)^), resulting on an expression of the volumetric expansion as function of time *t*. *RC* is the time constant and *P*_*i*_ is the maximum instantaneous pressure inside a module. We fitted both models to the experimental data (see model on Fig 3A and 3B) resulting in the following values for the different constants: Δ*V*_*τ*_ = 28.2%, *P*_*max*_ = 15.6 kPa for the static case and Δ*V*_*τ*_ = 28.2%, *P*_*max*_ = 138 kPa, *RC* = 2 ms, *P*_*i*_ = 137.9 kPa for the dynamic case. The later high level of pressure was only sustained during the very short transient. S2 Appendix explains both models and details the methodology used during these tests.

$$\Delta V_{chamber}\left(t\right)=\Delta V_{\tau }∙ln\left(\frac{P_{max}}{P_{max}-P_{i}\left(1-e^{\frac{-t}{RC}}\right)}\right)$$ (2)

### Adhesion

The soft modular units are able to attach to each other thanks to the magnetic force taking place between their faces. We measured the attraction force between a pair of facing magnets as function of distance and we fitted a quadratic model to the data. Equation S10 describes resulting model. S7 Fig shows the experimental data together with the fitted model. Detailed explanations of the measuring setup and model are presented in S3 Appendix.

**Fig 4:**
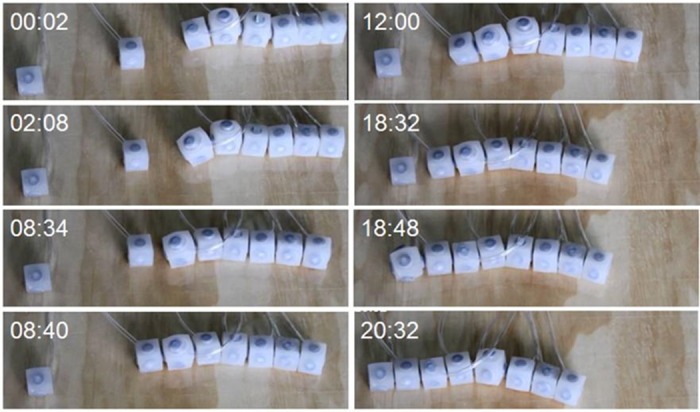
Cell adhesion during collective migration. A sequence of images displaying how a group of six connected modules is able to travel (from right to left) thanks to the coordinated inflation of modules. At time t = 8m:40s an adhesion occurs resulting in a new module incorporated to the group. The new seven-element robot continues travelling sideways until a new module adheres at t = 18m:48s. Then the resulting eight-element robot continues moving sideways.

Although the current locking mechanism still limits the scalability to only three modules suspending cantilever, several modules can be connected when supported over a horizontal surface or when floating on water. We tested the capability of a group of eight interconnected modules to purposively incorporate and attach new units while moving horizontally. Fig 4 shows a sequence of images displaying how a group of six connected modules is able to travel (from right to left) thanks to the coordinated inflation of modules. At time t = 8m:40s adhesion between the group and a new module occurs. The seven-element robot continues travelling along its own axis until a new module adheres at t = 18m:48s. The resulting eight-element robot continues moving. As the worm-like system incorporates new modules to itself, they are also incorporated to actuation by means of external coordination provided by a host computer and a motion capture system (Optitrack, http://www.optitrack.com).

### Collective Migration

**Fig 5:**
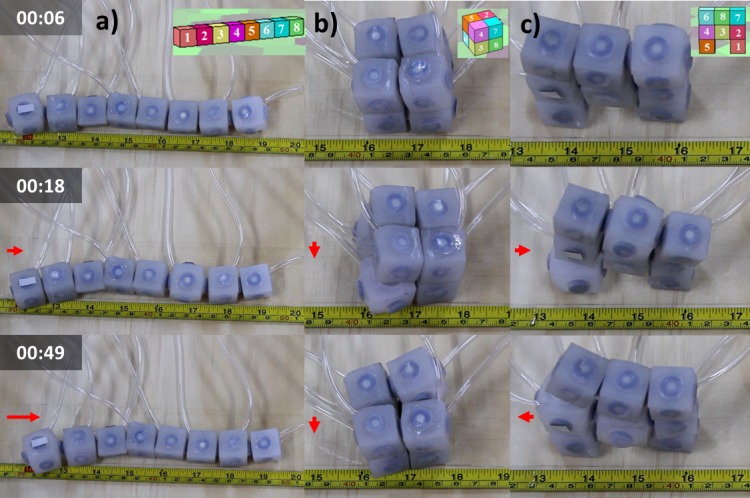
Instantaneous images taken during the locomotion of three different systems assembled using soft modular robotic units. a) System 1, a worm configuration, b) System 2, a 2×2×2 deformable cube c) System 3, a two legged machine. The timestamp is displayed on the left column in minutes:seconds format. Corresponding simulated systems are displayed on top. Red arrows indicate an estimated displacement vector.

**Fig 6:**
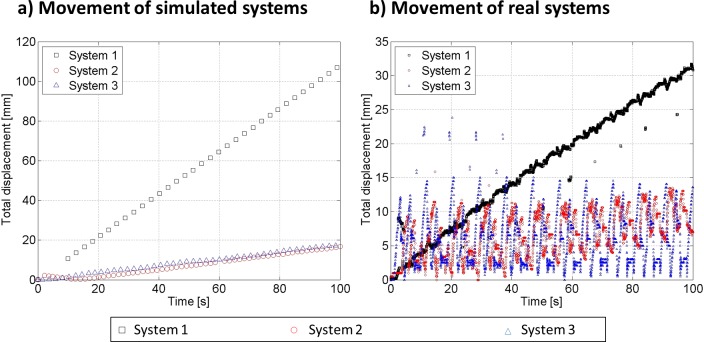
Characterization of locomotion. Displacement as function of time recorded from the three simulated (a) and real (b) systems shown in Fig 5. Experiments show consistency between the predicted behavior in simulation and measured behavior in reality. For example, system 1 consistently shows the ability to travel faster and at constant pace.

Inflation of a single isolated module does not result in any motion. Therefore we tested the locomotive capabilities of systems constructed using groups of soft modular robotic units. We measured the performance of these systems under simulation (VoxCad, http://www.voxcad.com) and then using real physical modules (see Fig 5). Details of the simulation implementation are presented in S4 Appendix. Sinusoidal volumetric sequences (Equation S11) commanded the inflation of simulated modules and binary sequences (Equation S12) commanded the inflation of real modules. Signals were shifted ¼ phase with respect to their immediate neighbor’s actuation command (See S1 Appendix for details). Measured displacements of all three systems are shown in Fig 6A for the simulated systems, and Fig 6B for their real physical implementation. Displacement of System 1, or the worm-like system, took place predominantly in the longitudinal direction and was consistently fastest. Inspection of video sequences (S1 Video) shows how peristaltic wave [27] propagation results in the overall displacement of the system. System 2, or compound cube, shows a small displacement toward the bottom. Finally, System 3, or legged, moves left and right without showing a noticeable total displacement. Despite speed magnitude differences observed between simulation and reality (Fig 6) one can appreciate how simulation allows predicting the overall trend of these systems.

### Delamination

**Fig 7:**
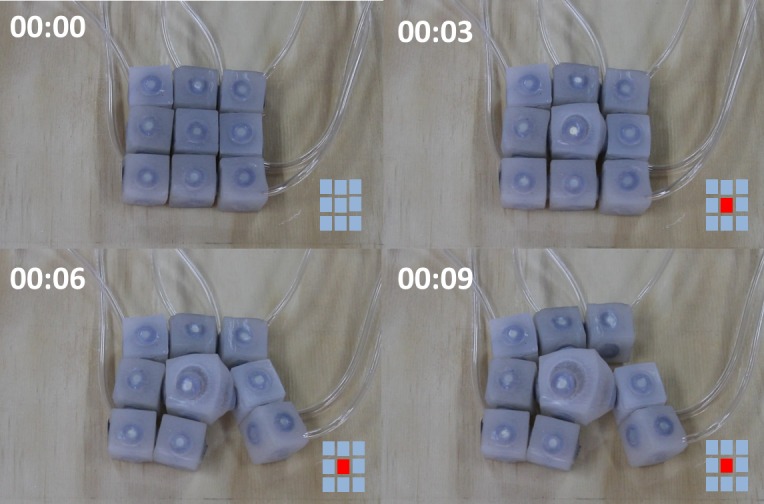
Cell Delamination. A sequence of images showing a behavior that resembles cell delamination on a modular system constructed with nine units. The sequence illustrates how inflation on a central module produces two detachments on the lower right portion of the image.

The ability to purposively detach modules is required to mimic the delamination capabilities of living cells and is widely recognized as a requirement for achieving self-reconfiguration. Usually providing a modular unit with actuation and control to enable detachment is expensive since modules should be embedded with the circuitry and mechanical actuators for docking/undocking (e.g. electro magnets). We found that coordinated inflation/deflation of soft neighboring modules can be used as an alternative method to detach specific areas of a soft modular assembly. Fig 7 shows a sequence of images taken while a group of nine units detaches certain elements thanks to the inflation of the central module. Despite the symmetry of the actuation pattern, detachments take place on the lower right portion of the structure. This symmetry break can be explained by small variations on the thickness of the silicone layer that covers magnets. This mode of detachment is a remarkable property of these soft modular robots and promises to become an alternative for discharging the onboard complexity of modular machines while enabling purposive localized detachment.

### Invagination and Involution

**Fig 8:**
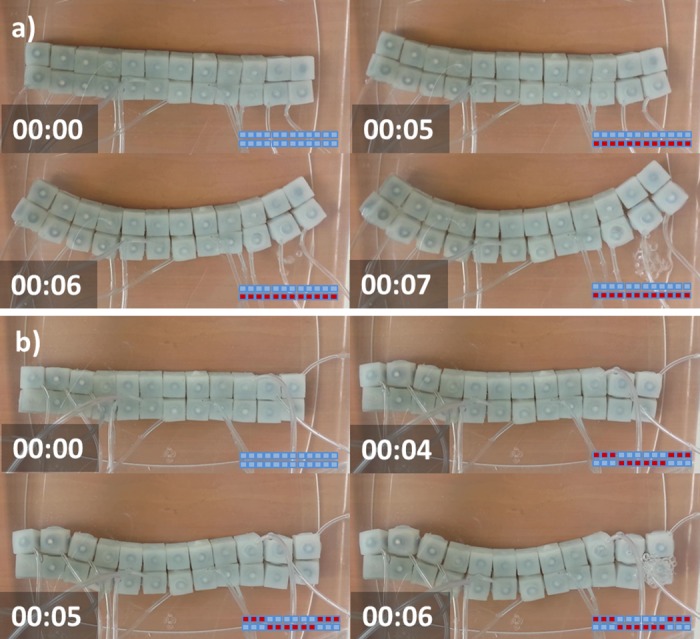
Cell invagination. Underwater experiments using a rectangular beam assembled using 24 soft modular robots. a) Transition from rectangular to curved shape. b) Transition from rectangular to double inflection curvature. Inflation patterns are displayed on a lower box using red for inflated modules and light blue for unactuated modules. Time stamps are in the format seconds:centiseconds.

We examined the potential of a group of 24 modules to reproduce the fundamental mechanics of cell invagination and involution. These behaviors require modular structures to flex, a process that can be reproduced by combining elements that expand while others dwindle. To reduce module-surface friction we performed this experiment, as well as the remainder tests, inside a vat containing water. The module buoyancy enabled lateral displacements while keeping structures submerged at the bottom of the recipient. We assembled a soft beam made of two parallel rows having twelve modules each, as shown in Fig 8. First we tested the capability of the system to flex by inflating the lower row elements and keeping the upper row unactuated (See Fig 8A). Then we tested the possibility of alternating the direction of curvature along the beam. We used the inflation pattern shown in Fig 8B where the first and last three elements of the upper row are inflated as well as the fourth up to the ninth element of the lower row. As a result, the assembly transitioned from a rectangular to a curvy shape that shows two curvature inflections. S4 Appendix shows an example of shapes that can be achieved when using eighty simulated modules. These behaviors demonstrate the potential of a group of soft modular robots to resemble to a great extend the cellular behaviors of invagination and involution.

### Epiboly

**Fig 9:**
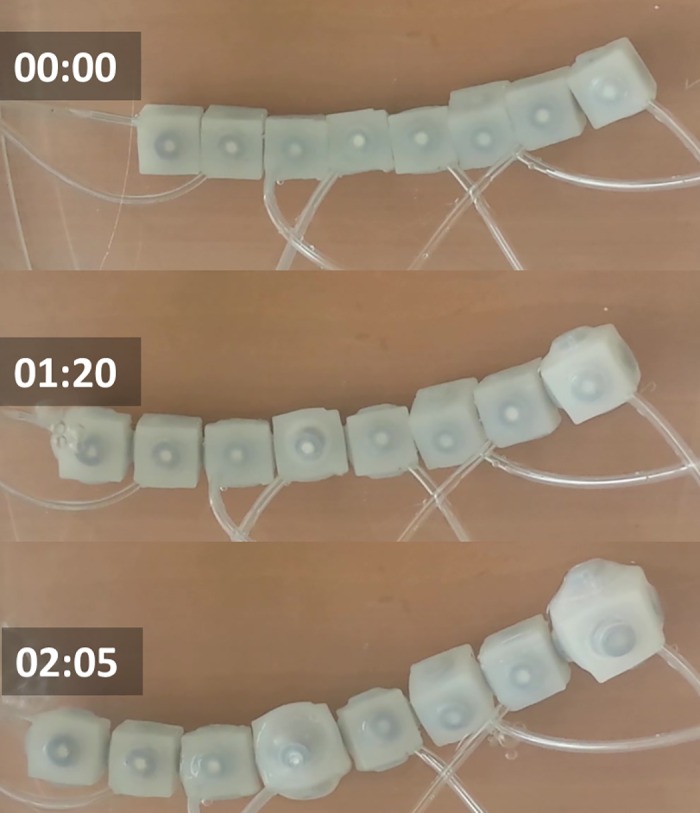
Cell Epiboly. Underwater experiments with a row of eight soft modules. The sequence of images displays a behavior that resembles the cellular behavior of epiboly. Initially modules are not pressurized. At t = 1s modules are pressurized resulting in 29% linear extension of the array at t = 2.05s. Time stamps are in the format seconds:centiseconds.

A simple experiment to replicate epiboly consisted on arranging eight modules on a row and then actuating over the arrangement to achieve linear extension. Fig 9 displays results from applying this procedure. Initially the group of modules is unactuated. At t = 1s the eight modules are pressurized (b) reaching an axial extension of 29% with respect to initial length at t = 2.05s.

### Self-Reconfiguration

We explored the ability of groups of soft modular robots to reconfigure themselves into different layouts. We built four modular setups by combining different number of actuated and unactuated modules. The remaining figures illustrate sequences of images displaying the inflation of modules and resulting reconfigurations for each setup. Each frame displays a time stamp together with a box illustrating the pattern of inflated modules in red and resting modules in light blue. The following sub-sections describe each configuration together with the experimental findings.

#### Setup 11-actuated, 11-unactuated (11A-11U)

**Fig 10:**
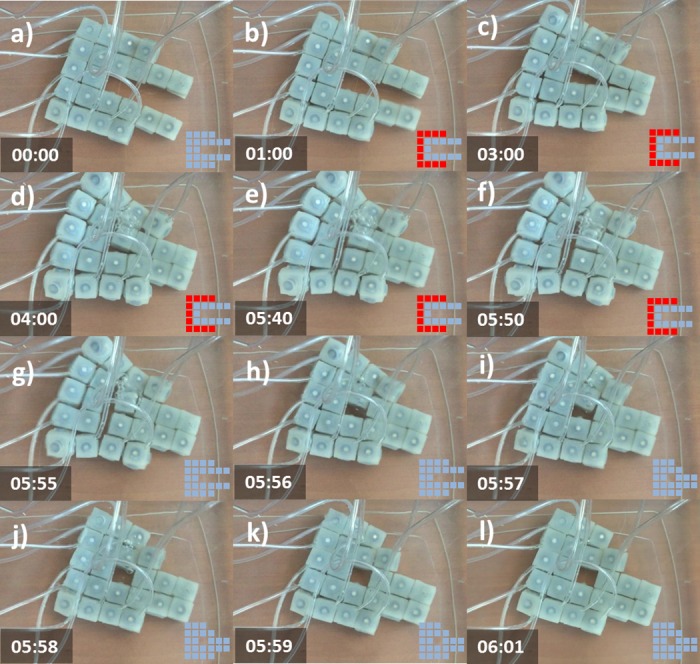
Self-Reconfiguration on configuration 11A-11U. Demonstration of self-reconfiguration on a group of 22 modules submerged in water. a) The group is initially configured in a ‘C’ shape. At t = 1s the outer modules are inflated (b) producing a closure of the shape (c). A delamination between four central modules takes place at t = 5.4s (e). Deactivation of peripheral modules results in migration of a group of four modules toward the center (h-i) resulting into a new stable configuration (j-l). Time stamps are in the format seconds:centiseconds.

Fig 10 displays a sequence of cell behaviors that enable the transition from an initial ‘C’ structure (a) to a final ‘O’ disposition (l). The reconfiguration is achieved by first producing a curvature on the exterior portion of the structure (b) by activating eleven modules located at the periphery. As a result form this behavior a group of modules adheres to their neighbors closing the ‘C’ into an ‘O’ (c). Then delamination takes place (e) producing a vertical streak on the center of the structure. At this point the peripheral modules are deactivated resulting in the migration of a group of four modules toward the center (h-i). This results in a new stable configuration (j-l).

#### Setup 2-actuated, 14- unactuated (2A-14U)

**Fig 11:**
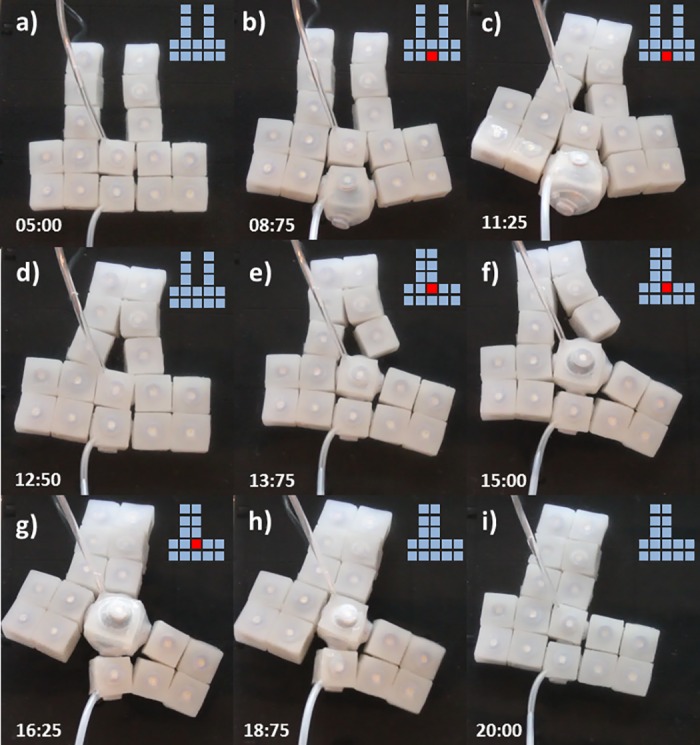
Self-Reconfiguration on setup 2A-14U. Demonstration of self-reconfiguration on a group of 16 modules submerged in water. Initially a lower-center module is inflated (b) resulting in the closing of the ‘C’. Subsequently the module deactivates (d). Then the upper-center module activates (e) and deactivates (h) resulting in the new stable ‘T’ shape (i). Time stamps are in the format seconds:centiseconds.

Fig 11 displays a sequence of cell behaviors that enable the transition from an initial ‘C’ structure (a) to a final ‘T’ disposition (i). First the lower-center module is inflated (b), this results in the closing of the ‘C’ with the transition of two modules toward the left. The module deactivates (d). Next the upper-center module activates (e) and deactivates (h) resulting in the new stable ‘T’ shape (i).

#### Setup 3-actuated, 13- unactuated (3A-13U)

**Fig 12:**
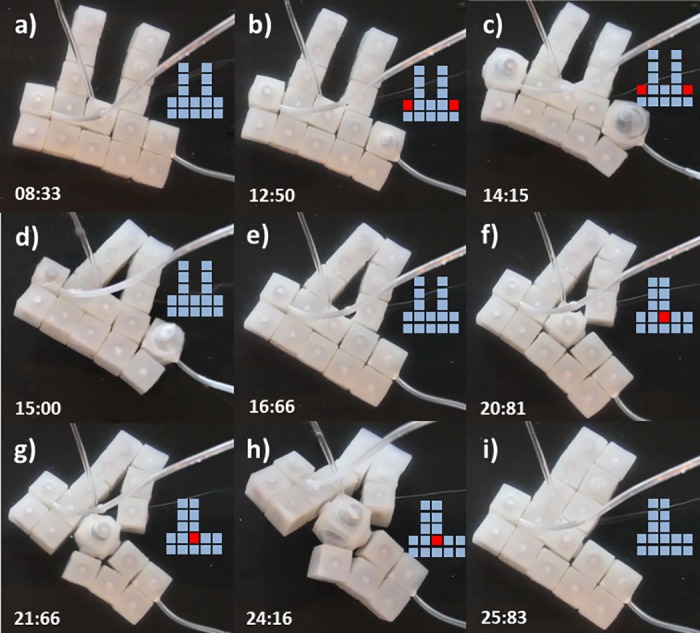
Self-Reconfiguration on setup 3A-13U. Demonstration of self-reconfiguration on a group of 16 modules submerged in water. The two lateral modules are first inflated (b) resulting in the closure of the ‘C’. Lateral modules are then deactivated (d). Next the upper-center module activates (f) and deactivates (i) resulting in the new stable ‘T’ shape.Time stamps are in the format seconds:centiseconds.

Fig 12 displays an alternative actuation sequence that results in equivalent topological change as previous situation (Transition from ‘C’ to ‘T’). In this case actuation takes place on two lateral and one central module. First the two lateral modules are inflated (b) resulting in the closure of the ‘C’ toward the center. Then the lateral modules are deactivated (d) and the upper-center module activates (f) and deactivates (i) resulting in the new stable ‘T’ shape (i).

#### Setup 2-actuated, 6-unactuated (2A-6U)

**Fig 13:**
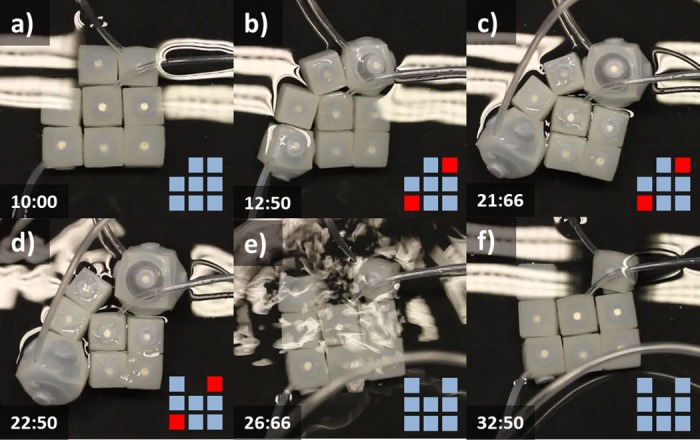
Self-Reconfiguration on setup 2A-6U. Demonstration of self-reconfiguration on a small group of 8 modules submerged in water. Two diagonally opposite modules inflate (b) producing the anti-clockwise rotation and translation of the upper-center module toward the left. At the same time the left most center module moves upside right and rotates clockwise resulting in a new inter-module attachment (c) and subsequent detachment (d). Finally a new stable squared shape emerges (e-f). Time stamps are in the format seconds:centiseconds.

Fig 13 displays a case of reconfiguration achieved with a reduced number of modules. Eight modules were initially configured into a square missing one of its vertices (a). Two diagonally opposite modules inflate (b) producing the anti-clockwise rotation and translation of the upper-center module toward the left. Simultaneously the left most center module moves upside right and rotates clockwise resulting in a new inter-module attachment (c) with subsequent detachment (d). Finally modules are deactivated and a new stable squared shape emerges (e-f).

#### Setup 2-actuated, 17-unactuated (2A-17U)

**Fig 14:**
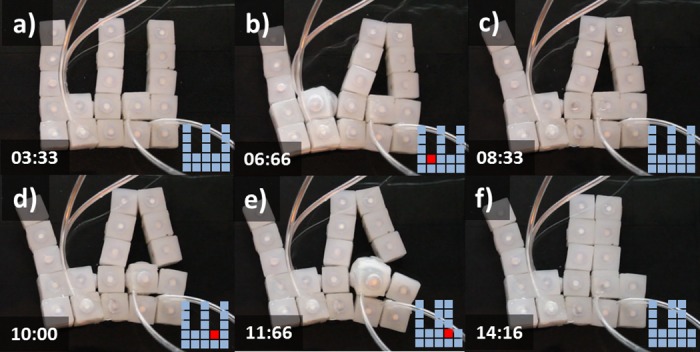
Self-Reconfiguration on setup 2A-17U, First Actuation Pattern. First part demonstrating how varying actuation pattern results in different configurations. A group of 19 modules is initially configured into an ‘E’ shape (a). In this case the lower-left module is first inflated (b) and then the lower-right module is inflated (d,e). This actuation pattern results in a final ‘F’ shape. Time stamps are in the format seconds:centiseconds.

**Fig 15:**
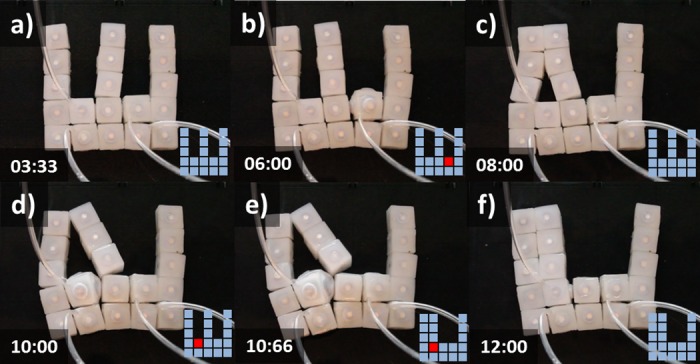
Self-Reconfiguration on setup 2A-17U, Second Actuation Pattern. Second part demonstrating how varying actuation pattern results in different configurations. A group of 19 modules is initially configured into the same ‘E’ shape (a) as in Fig 14A. In this case the lower-right module is first inflated (b) and then the lower-left module is inflated (d,e). This inverted actuation pattern results in a different ‘C’ shape. Time stamps are in the format seconds:centiseconds.

In this case we investigated the possibility of achieving different output configurations by keeping the same initial setup and just modifying the actuation pattern. Fig 14 displays an initial ‘E’ configuration (a). Actuation on a lower-left module (b) results in the partial displacement of one column from the right toward the center (c). The displacement is then consolidated by the actuation of a lower-right module (d,e) which results in the final ‘F’ configuration. Fig 15A displays the same ‘E’ initial configuration as shown on Fig 14A. In this case the actuation order is inverted and the lower-right module is first actuated (b). This results in the partial displacement of the central column toward the left (c). The displacement is then consolidated by the actuation of the lower-left module (d,e) which results in a different final ‘C’ configuration.

## Conclusion

This research demonstrates how simple pneumatically actuated soft modular robots can achieve self-reconfiguration and mimic various cell behaviors observed during morphogenesis. The fabrication method uses soft lithography for producing composite elastomeric hollow cubes and permanent magnets as passive docking mechanism. Instead of relying on rigid onboard-actuated docking mechanisms, we exploit the coordinated inflation/deflation of modules as a mechanism to detach and rearrange the position of specific modules. While traditional approaches to modular robotics would suggest the need of embedding modules with active latching mechanisms we observe how reconfiguration of units can be obtained thanks to the coordinated inflation of modules. Previous studies have suggested forms of modular reconfiguration on rigid heterogeneous robots without relying on active latching mechanisms [28]. Our results demonstrate that reconfiguration can be achieved with passive latching on even simpler homogenous modular robots, without having recourse to sophisticated mechanical implementations.

Departing from the same initial configuration we showed how different final configuration states can be consistently achieved when applying different control patterns.

While initial and final configurations are compatible with a rigid lattice we observe that self-reconfiguration is in general achieved thanks to the capability of soft modules to break a rigid lattice and sustain stable intermediate configurations that are only possible due to their elastic nature. A thorough understanding of this self-reconfiguring process requires modeling the details of the magneto-elastic interaction between modules, which is beyond the scope of the present study.

We also demonstrated how collective migration can be accomplished by a group of soft modules. In this case modules are able to migrate in the same direction while maintaining their inter-module connections. Similarly as in the case of living cells [29][30] we also verify that modules migrate more efficiently in groups rather than by themselves.

Our experiments also showed how simple soft modules can reproduce to great extend the overall mechanics of cell delamination, invagination, involution and epiboly. While other cell behaviors still remain unexplored (ingression, intercalation, convergent extension, etc.) our results already expose an interesting avenue for producing inexpensive, yet functioning, synthetic morphogenetic systems.

## Supporting Information

S1 Appendix Actuation of soft robotic modules. (PDF)

S2 Appendix Modeling the expansion of soft modular robotic cubes. (PDF)

S3 Appendix Characterization of inter module connection strength. (PDF)

S4 Appendix Simulation of soft modular robotic cubes. (PDF)

S1 Fig Pneu-electic diagram displaying the connections used for modulating the internal pressurization of soft modules. The air line of each soft module is driven by an independent pneu-electric circuit. Each circuit contains a diaphragm compressor for pressurization and a solenoid valve for relief, each one is activated using a transistor driven by 5v digital signals produced by an Arduino board.
(TIFF)

S2 Fig A simulation of eighty modules reproducing a double inflection behavior that resembles invagination. Inflated modules are displayed in blue and unactuated modules are shown with green. Time stamps are in the format seconds:centiseconds.
(TIFF)

S3 Fig Photos taken during the fabrication of one soft module. 1. Filling molds with silicone. 2. Taking parts out from the mold. 3. Cutting residual material. 4–6. Gluing together different parts. 7. Inserting magnet inside the frame. 8. Module showing one magnet sub-assembly inside.
(TIFF)

S4 Fig The actual pneumatic setup displaying solenoids, pneumatic pumps and lines. (TIFF)

S5 Fig Illustration of an unactuated empty module (left) and a heavily pressurized module (right) displaying a volumetric expansion 700% superior to initial volume. (TIFF)

S6 Fig Illustration of the setup used for measuring volumetric expansion of modules. a) Power source. b) Air pump. c) Manometer. d) Beaker. E) Module under water.
(TIFF)

S7 Fig Attraction force vs distance measured for a pair of magnets (Duramag 3000 Gauss NdFeB Neo magnet). The figure also displays the model (Equation S10) fitted to the data.
(TIFF)

S8 Fig a) Attraction force measurement setup. Distance was modulated by adding paper sheets (0.1mm thk) between magnets. The force was measured as the resulting weight required to detach magnets. Adding drops of water to the canister served to increase weight. b) Vertical arrangement of modules. c) Cantilever arrangement of modules.
(TIFF)

S9 Fig Images taken during the rapid volumetric response to an impulse of 137.9kPa. Time stamps are in milliseconds.
(TIFF)

S1 Video Experiments of Self-Reconfiguration, Invagination and Locomotion. (MP4)

S1 File Supporting CAD. (ZIP)
