# Supplementary material for: Soft Modular Robotic Cubes: Toward Replicating Morphogenetic Movements of the Embryo
Source: PLoS One. 2017 Jan 6;12(1):e0169179. doi: 10.1371/journal.pone.0169179 (PMC5218564; doi:10.1371/journal.pone.0169179)
Supplement: S1 Appendix — (PDF) [file pone.0169179.s001.pdf]

S1 Appendix: Actuation of soft robotic modules

Computer controlled inflation of each soft module was achieved thanks to the use of miniature pneumatic solenoid valves (12v, 26 kPa max pressure, normally closed) acting in series with air lines feed by miniature diaphragm air compressors (Thinker, 60 kPa pressure, 60 mL/min air flow). S1 Fig displays an electric-pneumatic diagram that illustrates the connections used for commanding twelve modules. S4 Fig displays the actual setup. Activation of each micro compressor or solenoid valve was triggered by an Arduino Leonardo. The low current signals were then amplified thanks to the use of transistors as displayed in the diagram.

Equation S11 shows the actuation signal $s_i^s(t)$ used for commanding the inflation of each simulated module i (with $i=\{1,..,8\}$ is a module index along the modular arrangement) at time t. Where f = 0.5 Hz and the phase $\varphi_i = \frac{i\pi}{2}$. Equation S12 shows the actuation signal $s_i^r(t)$ used for commanding the actuation of real modules. The later equation uses the $sign$ function to extract the sign of the sinusoid and deliver a binary output to the valves.

$$s_i^s(t) = \frac{1}{2} + \frac{1}{2}\sin(2\pi \mathrm{ft} + \varphi_i) \tag{S11}$$

$$s_i^r(t) = \frac{1}{2} + \frac{1}{2}sign(\sin(2\pi \mathrm{ft} + \varphi_i)) \tag{S12}$$

S1 Fig. **Pneu-electic diagram displaying the connections used for modulating the internal pressurization of soft modules.** The air line of each soft module is driven by an independent pneu-electric circuit. Each circuit contains a diaphragm compressor for pressurization and a solenoid valve for relief, each one is activated using a transistor driven by 5v digital signals produced by an Arduino board.
