# Supplementary material for: Soft Modular Robotic Cubes: Toward Replicating Morphogenetic Movements of the Embryo
Source: PLoS One. 2017 Jan 6;12(1):e0169179. doi: 10.1371/journal.pone.0169179 (PMC5218564; doi:10.1371/journal.pone.0169179)
Supplement: S2 Appendix — (PDF) [file pone.0169179.s002.pdf]

S2 Appendix:

Modeling the Expansion of Soft Modular Robotic Cubes

In order to understand the influence of pressure, flow, hose diameter and strain capacity in the dynamics of the actuation of a module, we modelled the resistance and capacitance of the fluidic system conformed by air pump, hose and silicon cube.

Hydrodynamic resistance

The flow **resistance** for a tube, or hydrodynamic resistance, generates a force acting opposite to the direction of the air flow and depends on the tube geometry, as shown in the following equation:

$$R = \frac{8\eta L}{\pi r^4}$$

, where η is the viscosity of the flow, L is the length of the tube, and r is the radius of the tube. The flow resistance generates a pressure drop along the tube according to the following equation:

$$P_{drop} = Rq$$

, where q corresponds to the volumetric flow rate of the fluid. The tube in our system is the hose. Hence, we can model the pressure drop from the air pump to the cube as follows:

$$P_i - P_o = Rq \tag{S1}$$

, where P_i corresponds to the pressure generated by the pump, P_o corresponds to the pressure that appears at the interior of the cube, and R is the flow resistance of the hose. Notice that the larger the diameter of the hose, the smaller the resistance. Also, the larger the length of the hose, the larger the resistance.

Hydrodynamic capacitance

The hydrodynamic **capacitance** in a fluidic system is a parameter that describes the change in the volume of a fluid, enclosed in a channel or chamber, in front of a change in pressure. This phenomenon occurs due to the compressibility of the fluid or the elasticity of the chamber.

Compressibility of a fluid

The compressibility of a fluid is measured by:

$$K_{fluid} = -\frac{1}{V}\frac{\partial V}{\partial P}$$

, where V is the initial volume of the fluid (equals to the volume of the chamber), ∂V the change in the volume of the fluid, and ∂P the change in the pressure of the fluid. The relation has negative sign because an increment on the pressure applied to a compressible fluid results in a reduction in its volume. The compressibility of the air is $K_{air} = \frac{1}{bar}$. Thus, the flow rate entering a chamber of volume V, because of fluid compression $\frac{d\Delta P}{dt}$, is:

$$q = -\frac{dV}{dt} = V\left(-\frac{1}{V}\frac{\partial V}{\partial P}\right)\frac{d\Delta P}{dt} = K_{fluid}V\frac{d\Delta P}{dt}$$

, and the hydrodynamic capacitance of the fluid is therefore:

$$C_f = K_{fluid}V.$$

Dilatability of a channel or a chamber

The dilatability of a channel or a chamber is defined by:

$$K_{chamber} = \frac{1}{V_{chamber}}\frac{\partial V_{chamber}}{\partial P}$$

, where $V_{chamber}$ corresponds to the initial volume of the chamber, $\partial V_{chamber}$ the change in the volume of the chamber, and ∂P the change in the pressure of the fluid. It has positive sign because an increment in pressure results in an increment on the volume of the chamber. Assuming a uniform pressure inside the chamber, the flow rate inflating the chamber is calculated as follows:

$$q = \frac{dV_{chamber}}{dt} = K_{chamber} V_{chamber} \frac{\Delta P}{dt}$$

, and the hydrodynamic capacitance of the chamber is therefore:

$$C_c = K_{chamber} V_{chamber}.$$

Notice that the volume of the cube, the strain capacity of the silicone and the compressibility of the air defines the hydrodynamic capacitance of the system. For example, the larger the strain capacity, the larger the hydrodynamic capacitance of the cube. The lower the compressibility of the fluid, the lower the capacitance of the system.

Hydrodynamic capacity of the system

Our fluidic system presents both characteristics: compressible fluid (air) and elastic chamber. Hence, following the analogy between electronics and fluidic systems, the total hydrodynamic capacitance of the system is:

$$C = C_f + C_c$$

, and the final dynamic response of the flow rate inflating the module is:

$$q = C \frac{d\Delta P}{dt}. \tag{S2}$$

Dynamic response of the pressure inside a module

To understand the dynamics of the pressure developed inside a module, P_o , we assume that its initial pressure is the atmospheric pressure and that the hose is not elastic. To continue, P_o corresponds to the pressure generated at the air pump minus the pressure drop along the hose, as described by Eq S1 rearranged as follows:

$$P_o = P_i - Rq. \tag{S3}$$

However, as the flow q varies as the module fills, we must replace Eq S2 in Eq S3 as follows:

$$P_o = P_i - RC\frac{dP_0}{dt}$$

, which implies that:

$$RC\frac{dP_0}{dt} + P_o = P_i. \tag{S4}$$

Eq S4 constitutes a first order system whose solution for P_o is given by the following expression:

$$P_o = P_i\left(1 - e^{-\frac{t}{RC}}\right). \tag{S5}$$

Eq S5 shows that the time constant of the system is $\tau_1 = RC$; the larger the time constant, the slower the step response of the system. Also, we know that geometry of the hose affects R and that strain capacity of the silicone affects C.

Impact of the characteristics of the air pump

P_i is a characteristic of the pump and defines the maximum pressure that can be built inside a module. Nonetheless, maximum flow rate of the pump is another important characteristic since it determines the speed at which the maximum pressure is built. In the formulation of Eq S5 we assumed that the pump can

provide any required flow to build up P_i instantaneously, which is true as long as the maximum flow required by the module is below the maximum flow rate of the pump. The maximum flow rate required by the module is defined by the following equation:

$$q_{max} = \frac{P_i}{R}$$

, assuming that P_i is a gauge pressure; i.e., absolute pressure minus atmospheric pressure.

Dynamic response of the volumetric expansion of a module

When determining the hydrodynamic capacitance of the chamber, we just considered $\partial V_{chamber}$, which can be obtained with the initial and final volume of a module. Nonetheless, the dynamics of the volumetric expansion is also of interest, for example, to analyze the transient response of a group of connected modules undergoing actuation at different phases. To obtain a model, we first measured the volumetric expansion of a module that resulted when slowly increasing the pressure P_o, as shown in Fig. 3a. For each measurement point, volumetric expansion was measured after the system stabilized.

Although we measured in this experiment P_i (not P_o) at the gauge location (See S6 Fig), the volumetric expansion was measured once the system reached its stationary state, therefore $P_i = P_o$. To continue, Fig. 3a also shows that the volumetric response has an asymptote at the pressure: the maximum resistance of a module, P_{max}. With this in mind, we fitted a model to the measured volumetric response, as follows:

$$\Delta V_{chamber}(P_o) = \Delta V_\tau \cdot ln\left(\frac{P_{max}}{P_{max} - P_o}\right), \qquad \text{with } P_o < P_{max}. \qquad \text{(S6)}$$

, where ΔV_τ is the volumetric expansion of a module that triggers an accelerated volumetric expansion. As seen in Fig. 3a, the maximum pressure our modules can sustain is about $P_{max} \approx 15.6kPa$. Fig. 3a also displays the model shown in Eq S6 fitted to the data when considering $\Delta V_\tau = 28.2\%$. Resulting model fits

nicely the data (r=0.96). If we consider that volumetric expansion can be expressed as $\Delta V_{chamber} = 100\left(\frac{V(P)-V_0}{V_0}\right)$, Eq S6 can be expressed in terms of volume, as follows:

$$V(P_0) = (V_\tau - V_0) \cdot ln\left(\frac{P_{max}}{P_{max}-P_o}\right) + V_0, \tag{S7}$$

where V_0 is the initial volume of the cube, and $V_\tau = \Delta V_\tau \frac{V_0}{100} + V_0$ is the volume of the cube that triggers an accelerated volumetric expansion.

We can also find the volumetric expansion of a module as a function of time by replacing Eq. S5 into Eq. S6, as follows:

$$\Delta V_{chamber}(t) = \Delta V_\tau \cdot ln\left(\frac{P_{max}}{P_{max}-P_i\left(1-e^{\frac{-t}{RC}}\right)}\right), \tag{S8}$$

which can also be expressed in terms of volume, as follows:

$$V(t) = (V_\tau - V_0) \cdot ln\left(\frac{P_{max}}{P_{max}-P_i\left(1-e^{\frac{-t}{RC}}\right)}\right) + V_0. \tag{S9}$$

On a second experiment we measured the transient volumetric response of a module to an impulse. This was implemented by applying a large pneumatic pressure P_i = 138 kPa over a short period of time (nearly 50ms). Figure 3b shows the measured transient volumetric response together with the model of Eq S8. The model was fitted with the parameters: $P_i = (138 - \varepsilon)$ kPa, with $\varepsilon = 1 \times 10^{-13}$ being a very small numeric constant, $\Delta V_\tau = 28.2\%$, $RC = 2ms$, and $P_{max} = 138\ kPa$. The model fits nicely the data (r = 0.97). Due to the short period of time the module is able to sustain the high level of pressure without collapsing.

Experimental Setup for Characterizing Volumetric Response

Volumetric expansion vs pressure was experimentally measured with the setup shown in S6 Fig. A computer controlled power source (a) drives a miniature diaphragm air compressor (Thinker, 60 kPa pressure, 60 mL/min air flow) shown in (b). The compressor is linked by a hose to a pressure gauge (d) and a soft module submerged in water (c). The beak scale allows measuring volumetric variations as function of pressure changes. Resulting data is shown in Fig. 3b. An example of volume variation for unactuated vs heavily pressurized module is shown in S5 Fig.

Figure S5: Illustration of an unactuated empty module (left) and a heavily pressurized module (right) displaying a volumetric expansion 700% superior to initial volume.

A breakout expansion test was performed with a module inside the beaker filled with water. Volumetric expansion was also measured by observing water displacement as the compressor injected air through the hose. As a result, the modules expanded up to 1460% (125cc) before bubbles appeared indicating failure.

In order to determine the speed of reaction of the system, the step response of the volumetric expansion was registered. The magnitude of the pressure step was 137.9kPa. The instantaneous volume was estimated using video frames. The resulting measured expansion speed was rather high since the module reached its final volume after 200ms, as shown in Fig 3a**.**
