# Supplementary material for: Soft Modular Robotic Cubes: Toward Replicating Morphogenetic Movements of the Embryo
Source: PLoS One. 2017 Jan 6;12(1):e0169179. doi: 10.1371/journal.pone.0169179 (PMC5218564; doi:10.1371/journal.pone.0169179)
Supplement: S3 Appendix — (PDF) [file pone.0169179.s003.pdf]

S3 Appendix:

Characterization of inter module connection strength

We measured the attraction force between a pair of Neodymium cylindrical permanent magnets (Duramag 3000 Gauss NdFeB Neo magnet, 6mm dia × 2mm thk). The setup consisted on pulling with varying force one magnet suspended from another fixed magnet. Variable force was achieved by attaching a water filled canister to the suspended magnet. The distance between magnets was increased by adding paper sheets (0.1mm thk), one below the other, until a desired distance was accomplished. For each distance the force was measured by the resulting weight required to detach magnets. Weight was then incremented by adding drops of water to the canister. Resulting relationship is shown in S7 Fig. Least squares fitting to the data (R = 0.9979) resulted in the quadratic model shown in Equation S10, where *d* is the distance measured in millimeters and *F* is force measured in kgf.

Figure S7 Attraction force vs distance measured for a pair of magnets (Duramag 3000 Gauss NdFeB Neo magnet). The figure also displays the model (Equation S10) fitted to the data.

$$F = 0.074d^2 - 0.385d + 0.564 \tag{S10}$$

Additional tests were performed to measure connection strength as the number of modules that can be attached either in cantilever or vertical disposition (see S8 Fig b,c). For the first test, a module was fixed while subsequent modules were connected right below one another to form a vertical column. The second test was very similar, but subsequent modules were connected to a lateral face to form a horizontal cantilever row. Connection strength in both cases was measured

as the maximum number of modules that could stay connected before the structure collapsed. Modules were unactuated during these experiments.

Figure S8 a) Attraction force measurement setup. Distance was modulated by adding paper sheets (0.1mm thk) between magnets. The force was measured as the resulting weight required to detach magnets. Adding drops of water to the canister served to increase weight. b) Vertical arrangement of modules. c) Cantilever arrangement of modules.

The experiments indicated that up to eight modules can be connected vertically and up to three modules cantilever. The success of cantilever trials was sometimes affected by small variations on thickness of the silicone membrane covering the magnets of adjacent modules. Adhesion force between contacting modules was measured to be 0.08 kgf. This is achieved at the inter-magnet distance of 2 mm.
