# Supplementary material for: Soft Modular Robotic Cubes: Toward Replicating Morphogenetic Movements of the Embryo
Source: PLoS One. 2017 Jan 6;12(1):e0169179. doi: 10.1371/journal.pone.0169179 (PMC5218564; doi:10.1371/journal.pone.0169179)
Supplement: S4 Appendix — (PDF) [file pone.0169179.s004.pdf]

S2: Simulation of Soft Modular Robotic Cubes

VoxCad (http://www.voxcad.com) is a modeling and analyzing software for soft discrete volumetric elements that allows, among other functions, creating structures in space and provide them with volumetric actuation. Cubic-shaped voxels of 20x20x20 mm were used, modelled as linear deformation material with an elastic modulus = 1 MPa, Poisson's ratio=0.35, density=1070 kg m^{-3}, coefficient of thermal expansion = 0.1 °C^{-1}, static friction coefficient = 2.0 and dynamic friction coefficient = 1.0. Simulation temperature ranged from 25 °C (temperature where no expansion occurs) to 26 °C with a period of 1.04 s, with self-collision calculation enabled.

To mention few differences between reality and simulation one can consider that real modules are joined by a small area on the center of their faces while simulated modules consider bonding over all the face surface. Real expansions are not completely homogeneous and modules acquire spheroidal shapes rather than cubic when inflated.

Given these promising results we further exploited simulation to test the capability of larger groups of modules to reproduce some complex cell behaviors. S2 Fig displays a simulated soft modular behavior that resembles invagination when using eighty modules. Inflated modules are shown in blue while unactuated modules are displayed with green. One can observe how the coordinated actuation of modules serve to produce the alternating curvatures observed during this cellular behavior.

S2 Fig. A simulation of eighty modules reproducing a double inflection behavior that resembles invagination. Inflated modules are displayed in blue and unactuated modules are shown with green. Time stamps are in the format seconds:centiseconds.
