# Supplementary figures and images for: Soft Modular Robotic Cubes: Toward Replicating Morphogenetic Movements of the Embryo
Source: PLoS One. 2017 Jan 6;12(1):e0169179. doi: 10.1371/journal.pone.0169179 (PMC5218564; doi:10.1371/journal.pone.0169179)

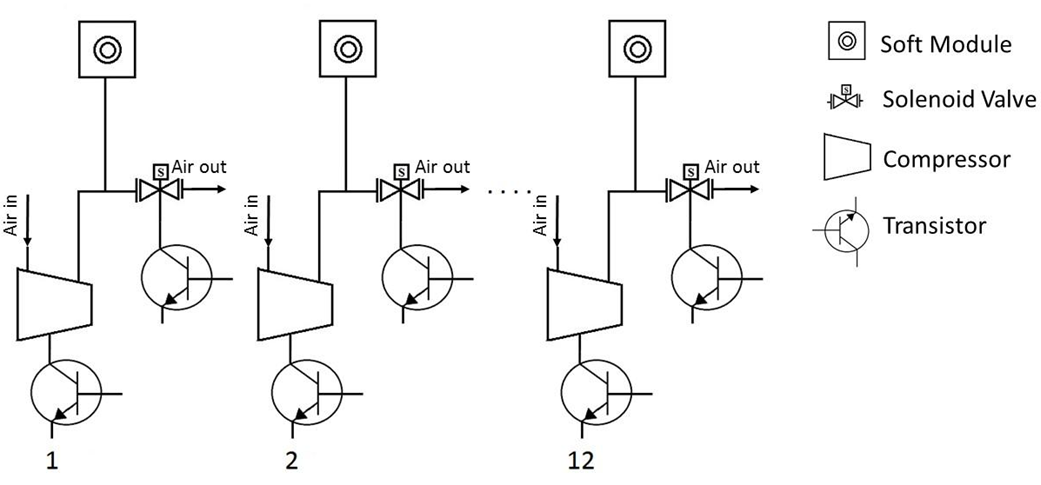

Supplement: S1 Fig — The air line of each soft module is driven by an independent pneu-electric circuit. Each circuit contains a diaphragm compressor for pressurization and a solenoid valve for relief, each one is activated using a transistor driven by 5v digital signals produced by an Arduino board. (TIFF) [file pone.0169179.s005.tiff]

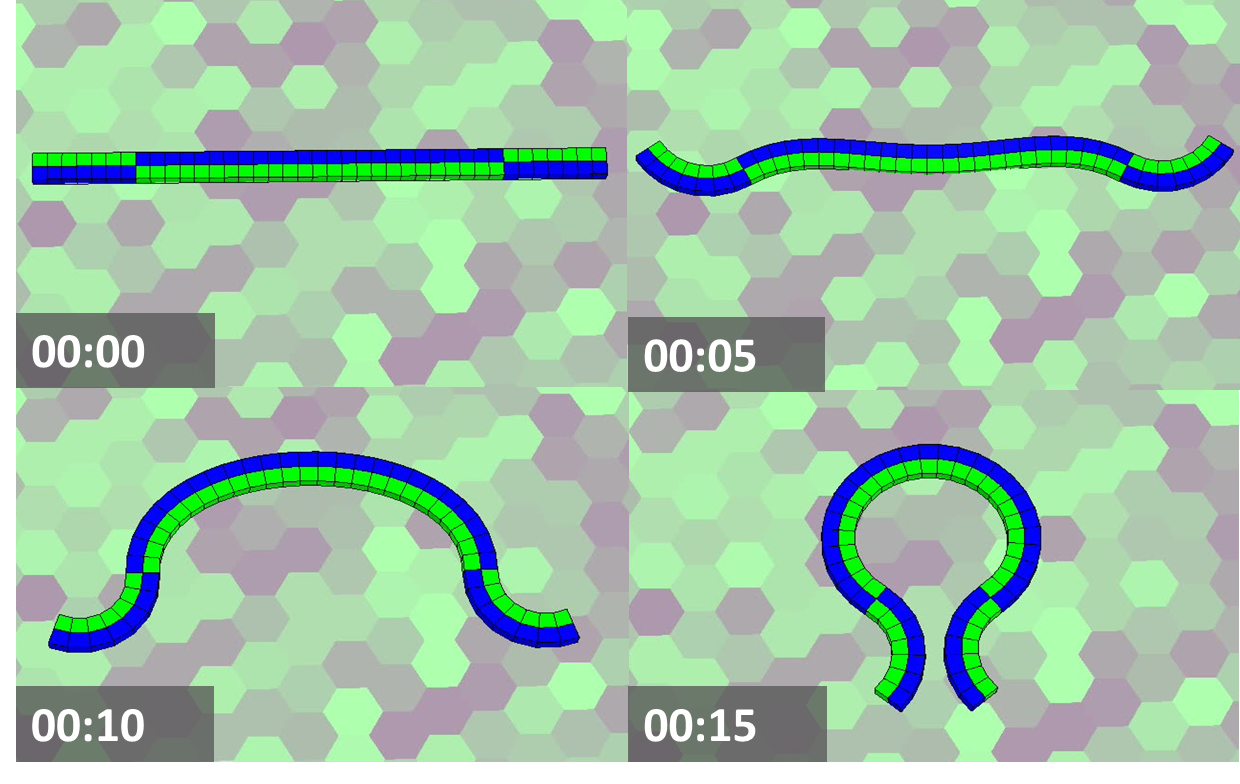

Supplement: S2 Fig — Inflated modules are displayed in blue and unactuated modules are shown with green. Time stamps are in the format seconds:centiseconds. (TIFF) [file pone.0169179.s006.tiff]

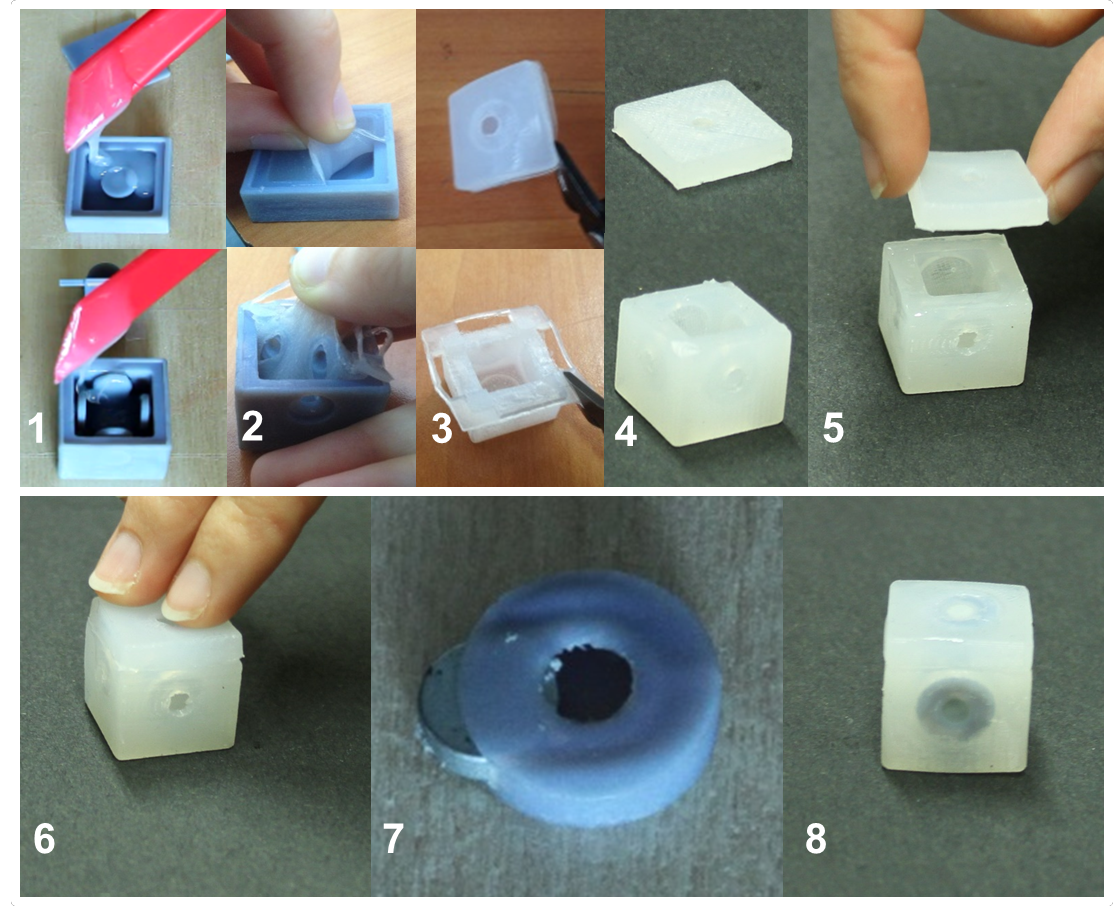

Supplement: S3 Fig — 1. Filling molds with silicone. 2. Taking parts out from the mold. 3. Cutting residual material. 4–6. Gluing together different parts. 7. Inserting magnet inside the frame. 8. Module showing one magnet sub-assembly inside. (TIFF) [file pone.0169179.s007.tiff]

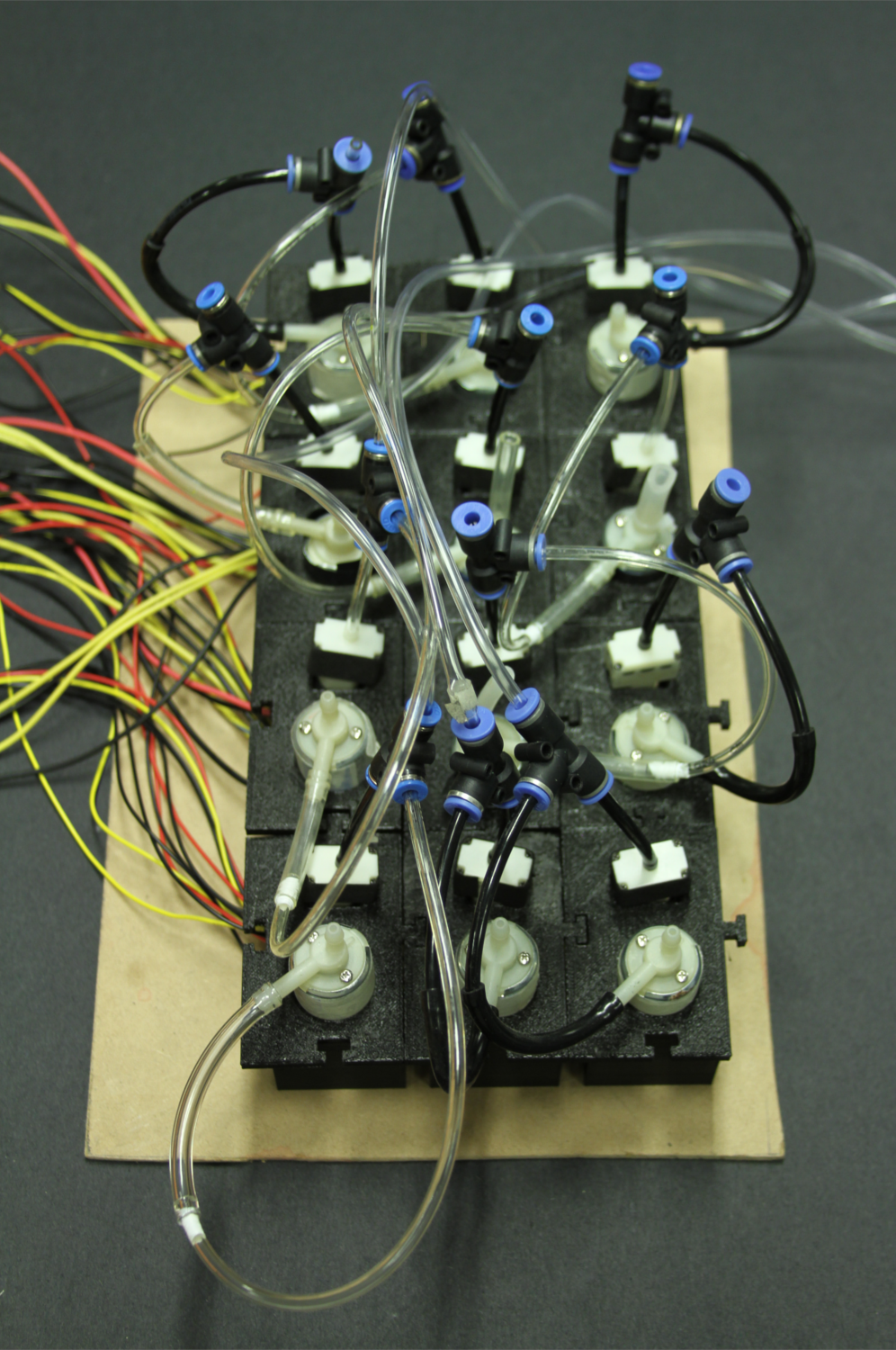

Supplement: S4 Fig — (TIFF) [file pone.0169179.s008.tiff]

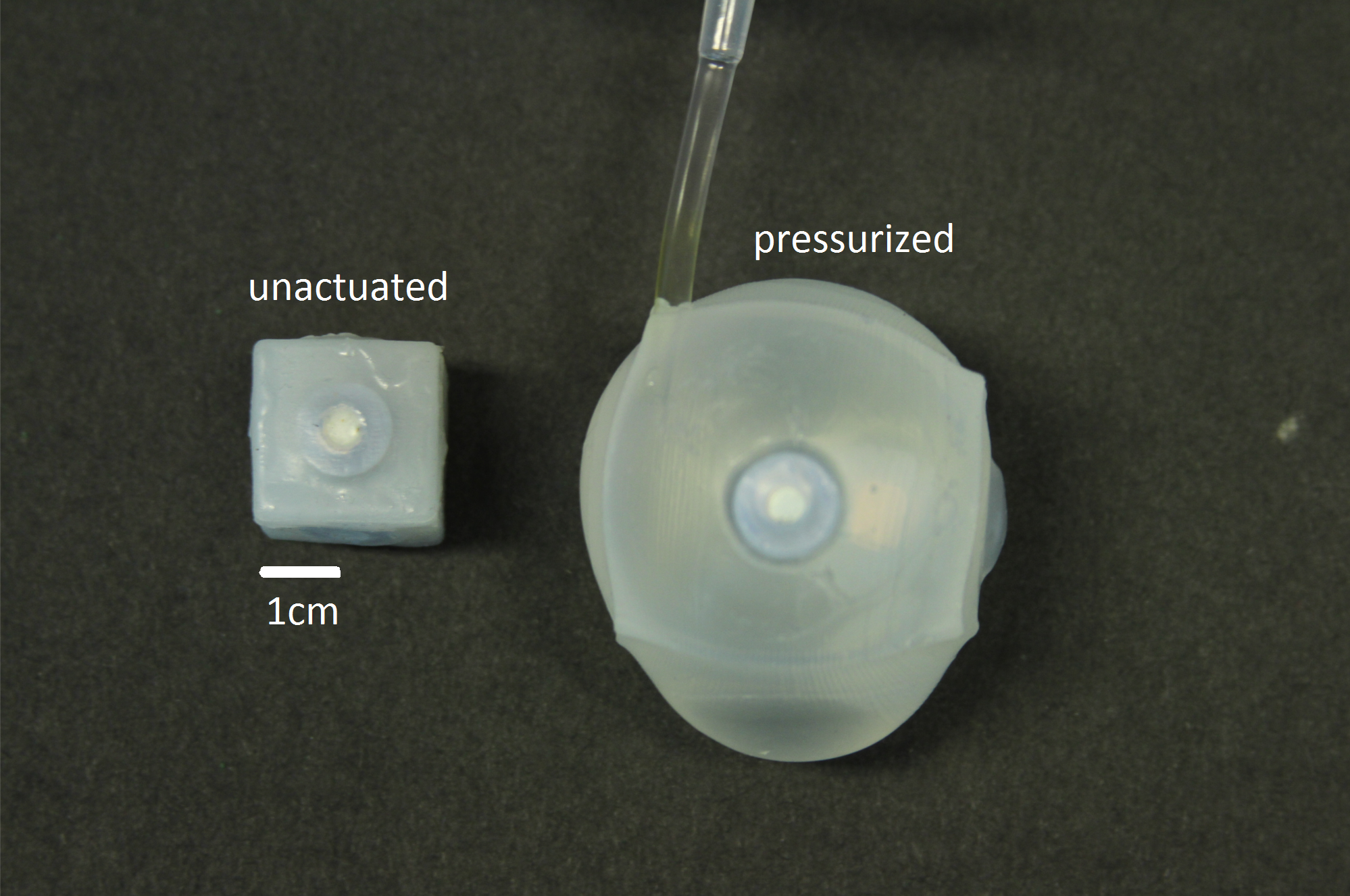

Supplement: S5 Fig — (TIFF) [file pone.0169179.s009.tiff]

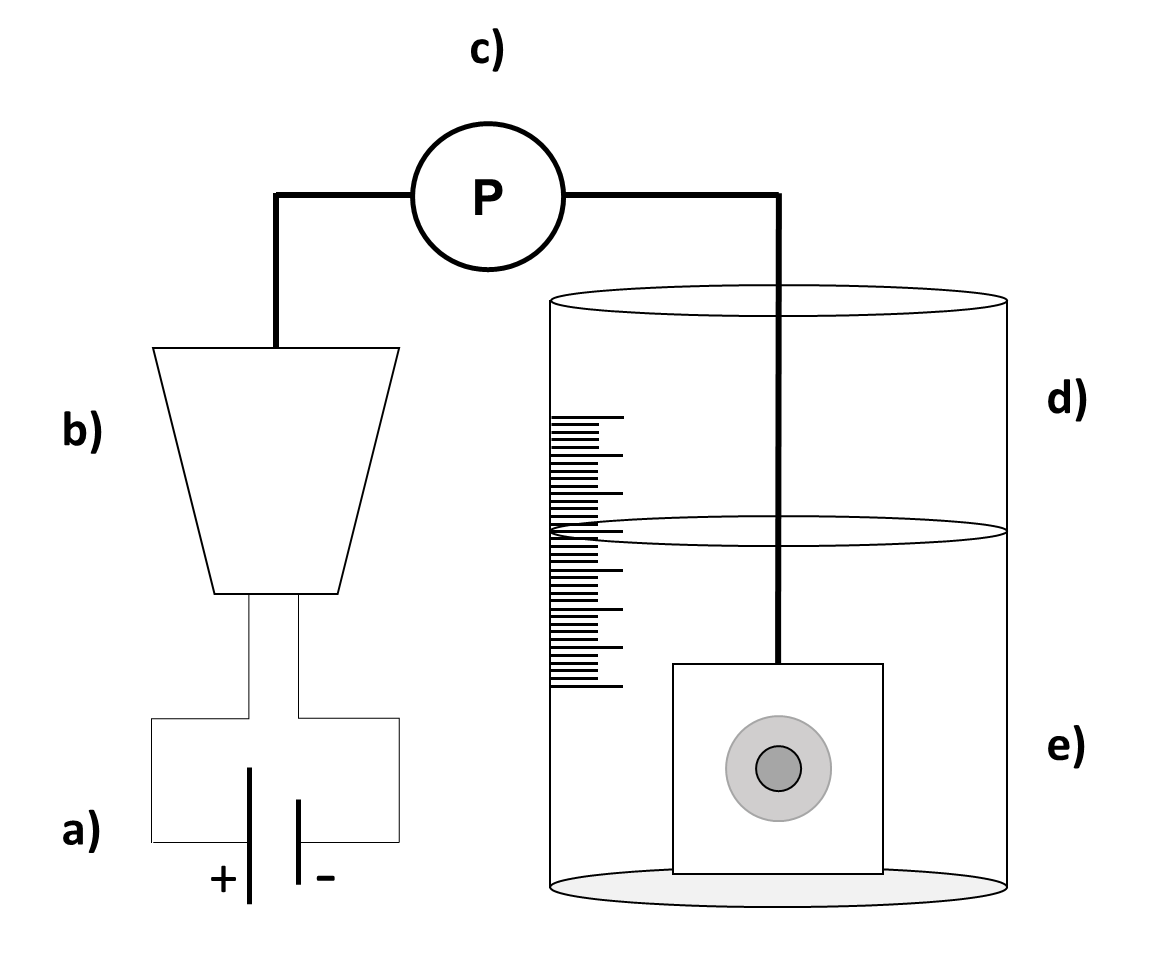

Supplement: S6 Fig — a) Power source. b) Air pump. c) Manometer. d) Beaker. E) Module under water. (TIFF) [file pone.0169179.s010.tiff]

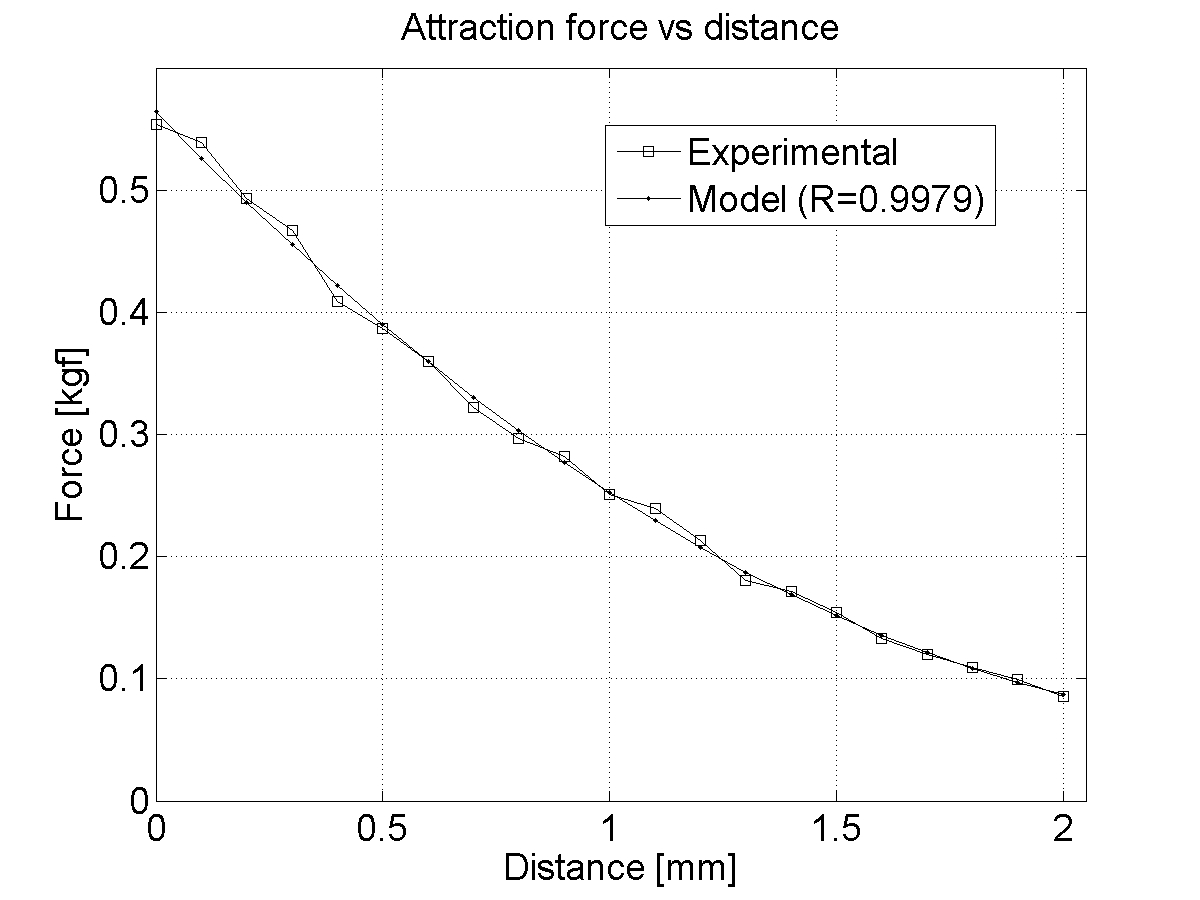

Supplement: S7 Fig — The figure also displays the model (Equation S10) fitted to the data. (TIFF) [file pone.0169179.s011.tiff]

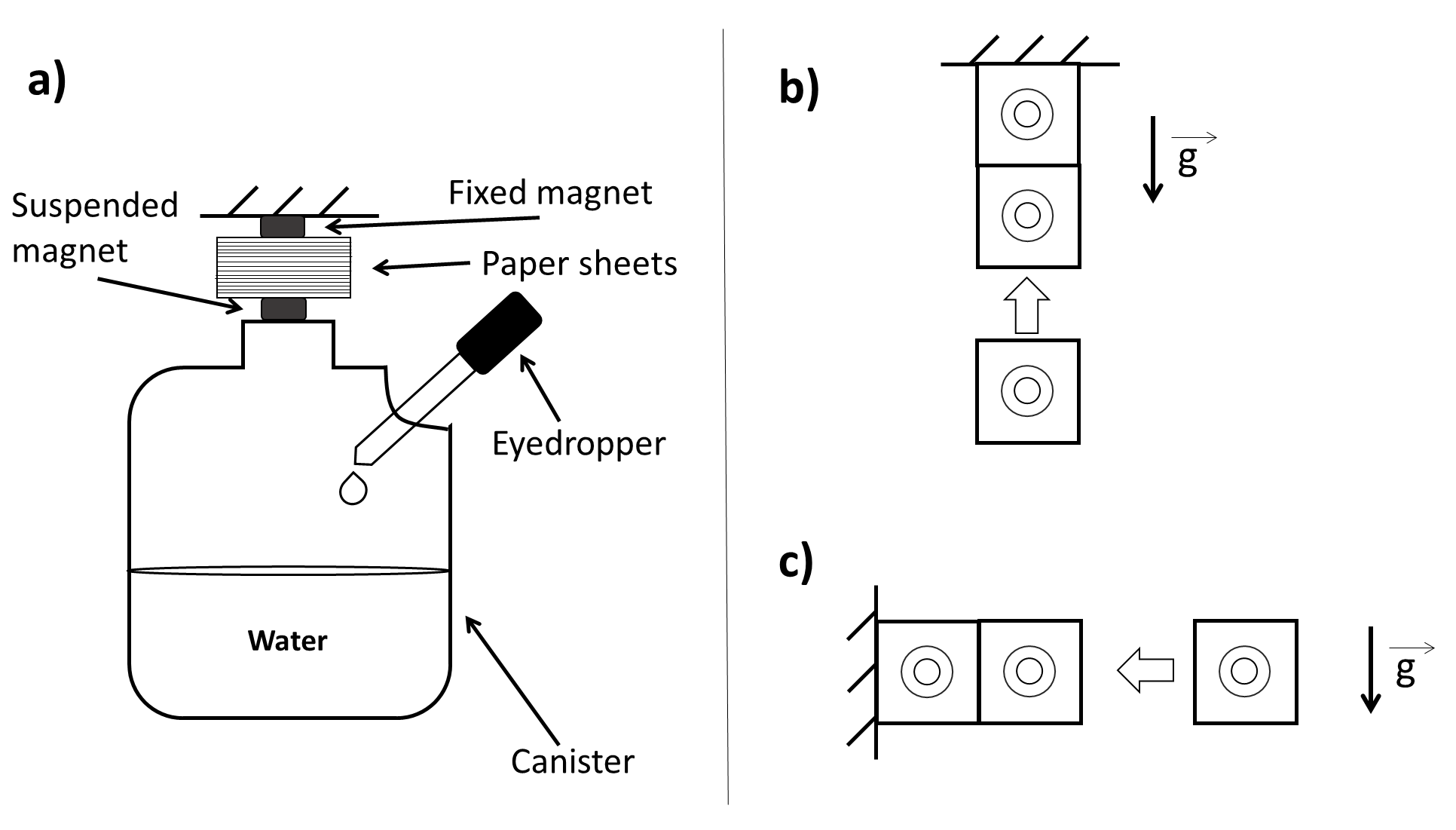

Supplement: S8 Fig — a) Attraction force measurement setup. Distance was modulated by adding paper sheets (0.1mm thk) between magnets. The force was measured as the resulting weight required to detach magnets. Adding drops of water to the canister served to increase weight. b) Vertical arrangement of modules. c) Cantilever arrangement of modules. (TIFF) [file pone.0169179.s012.tiff]

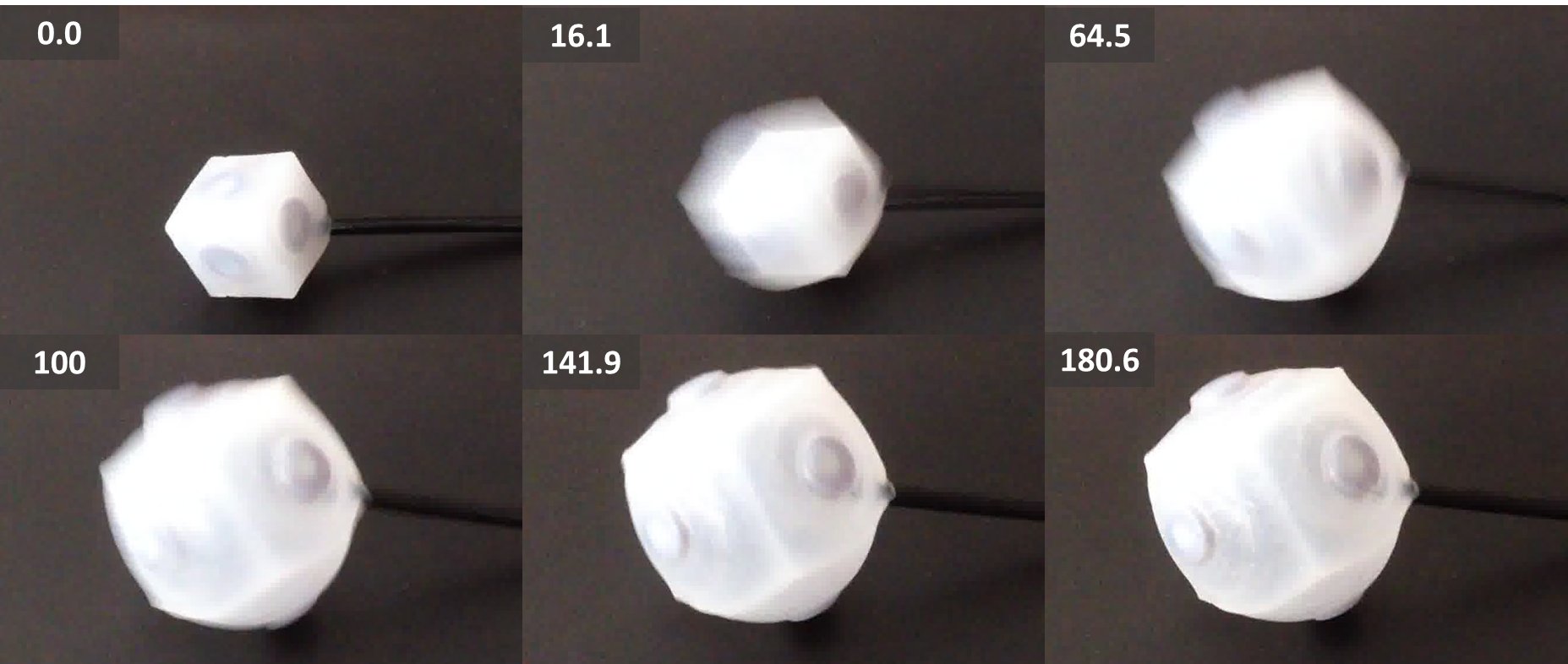

Supplement: S9 Fig — Time stamps are in milliseconds. (TIFF) [file pone.0169179.s013.tiff]
